# “Dental team-based education” Dental team perspectives and experiences about weight stigma: a qualitative analysis

**DOI:** 10.1186/s12903-025-05854-1

**Published:** 2025-04-06

**Authors:** Zanab Malik, Kate A. McBride, Kathryn Williams, Deborah Cockrell, Clare E. Collins

**Affiliations:** 1 School of Health Sciences (Oral Health), College of Health, Medicine and Wellbeing, The University of Newcastle, Ourimbah, NSW Australia; 2 South Western Sydney Local Health District, Oral Health Services, NSW Health, Campbelltown, NSW Australia; 3 Translational Health Research Institute, Western Sydney University, Penrith, NSW Australia; 4 School of Medicine, Western Sydney University, Penrith, NSW Australia; 5 Nepean Blue Mountains Family Metabolic Health Service, Nepean Hospital, Nepean Blue Mountains Local Health District, Kingswood, NSW Australia; 6 Charles Perkins Centre-Nepean, The University of Sydney, Sydney, NSW Australia; 7 School of Health Sciences (Nutrition and Dietetics), College of Health, Medicine and Wellbeing, The University of Newcastle, Callaghan, NSW Australia; 8 Food and Nutrition Research Program, Hunter Medical Research Institute, Newcastle, NSW Australia

**Keywords:** Weight stigma, Dental professionals, Dental team, Severe obesity

## Abstract

**Background:**

There is evidence from qualitative and quantitative studies of the presence of weight stigma in the dental setting from the patient perspective. However, the perspectives of various members of the dental team and their observations and experiences of weight stigma are unknown. Given dental specialists in Special Needs Dentistry (SND) are often referred patients living with severe obesity for dental management, their perspectives are of specific importance, particularly with respect to currently employed and recommended weight stigma reduction strategies.

**Aims:**

Our qualitative study aimed to identify and explore among dental professionals and support staff in Australia, their perspectives and experiences of weight stigma. We aimed to identify any currently employed, and recommendations for, stigma reduction from SND specialists.

**Methods:**

Focus groups and semi-structured interviews (*n* = 34 participants) were conducted with two groups; dental professionals and support staff from regional New South Wales; and SND specialists in Australia. Recordings were transcribed verbatim and analysed using thematic (inductive) analysis.

**Results:**

Key themes emerged related to observed experiences with weight stigma, with differing perspectives based on professional roles. The impact of weight stigma on dental management was highlighted. Stigma reduction strategies with an educational focus were also identified from SND specialist participants.

**Conclusion:**

The current study explored observations of weight stigma in various dental settings and perspectives which differed based on participant awareness and professional role. The negative impact of weight stigma on preventive dental discussions was identified. Stigma reduction strategies need to target the identified barriers and address the complex drivers of weight stigma before implementation. The findings of the current study emphasise the role for team-based education, led and guided by SND specialists through their professional advocacy roles and encourages the development of a dental team action plan to respond to observed experiences of weight stigma in the workplace.

**Supplementary Information:**

The online version contains supplementary material available at 10.1186/s12903-025-05854-1.

## Introduction

Weight bias and obesity related stigma is the negative stereotyping, prejudice, and unfair treatment experienced across many spheres of daily life by people living with obesity, including when accessing healthcare.[1] Weight stigma is complex and can be both implicit, such as automatic, negative attitudes, or explicit, where it is conscious or deliberate [2]. There is evidence of weight stigma in healthcare settings, where it has the potential to generate major health disparities and lead to avoidance of healthcare [3]. A key finding of a recently published scoping review identified weight stigma is also present among dental professionals and in the dental setting and may lead to poorer quality dental care [4]. Qualitative data from patients living with clinically severe obesity have also reported weight stigma as a barrier to accessing dental services in Australia [5]. The experience of weight stigma was a similar finding from qualitative data reported by a group of patients with obesity in the UK [6]. However, the experiences of weight stigma specifically from the dental team perspective and how this may impact on dental management is currently unknown.

In Australia, people living with obesity (body mass index (BMI) ≥ 30 kg/m^2^) receive oral healthcare services in public and private general dental settings. However, people living with severe obesity (BMI ≥ 40 kg/m^2^), specifically those with very high body weights, are frequently referred to public dental specialists in Special Needs Dentistry (SND) for comprehensive dental management necessitating the use of bariatric dental chairs [7, 8]. These chairs are indicated for use when the safe dental chair working limits are exceeded, and are predominantly located within tertiary dental hospitals within SND departments and few regional locations [7]. As such, SND specialists may have increased exposure to this cohort of patients with severe obesity and may have additional insights on the subject of weight stigma and education around this subject for the dental team.

This qualitative study aimed to explore the perspectives and experiences of weight stigma in the dental setting and, among the dental team, including SND specialists. Uniquely, this study also includes the perspective of support staff, as these team members are often the first contact any individual has with oral healthcare services.

## Materials and methods

Ethics approval was obtained from the Central Coast Local Health District Human Research Office (number 1122-101C). All participants provided their consent to participate. Participants included dental professionals (registered general dentists, oral health therapists) and support staff (dental assistants, dental receptionists) working in private and public regional practices in New South Wales and with access to a public based bariatric dental chair, and registered dental specialists in SND across Australia. Recruitment was via email to all public based employees within Central Coast Local Health District Oral Health Services and to registered dental professionals via the Central Coast Division mailing list obtained from the Australian Dental Association. For SND specialist participants, recruitment was via email to the Australian and New Zealand Academy of Special Needs Dentistry membership for Australian members only, of which there were 26 at time of recruitment. The email included a focus group invitation letter and participant information sheet explaining the background to the research and background of researcher ZM.

Invited participants who provided consent to participate in the focus groups were asked to sign a focus group confidentiality agreement form, to emphasize the need to maintain privacy of discussions and encourage participation. A semi-structured interview schedule (Supplementary Figure S1) was developed by a multidisciplinary project team (two dental specialists in SND and Oral Surgery, an academic nutritionist and an endocrinologist and obesity specialist) for both clinicians and support staff. The focus group interview schedule was piloted using two semi-structured interviews with clinicians (*n *= 2) and deemed to be appropriate. Focus groups were either carried out via an online platform (Microsoft Teams™) or in person in a neutral non-clinical location such as administrative office space.

Two series of focus groups were scheduled with consenting participants, with participants randomly allocated into groups with people of the same professional role (general dental professionals and support staff followed by SND specialists nationally). This was done using a lottery method for each group until at least 2–3 participants were allocated to a focus group based on their schedule availability. Data were collected between March and May 2023. Focus groups were conducted by ZM and moderated by KK. Field notes were made during and after each focus group by both ZM and KK. Data collection ceased once data saturation was reached, which is when sufficient information had been obtained to replicate the study and no new further information was being collected [9].

### Research team and reflexivity

ZM is a female dental specialist in SND who was working part time (2 days/week) in Central Coast Local Health District at time of data collection. ZM has experience in qualitative data collection and has clinical experience in the dental management of people living with obesity. This research was part of her PhD studies.

A female research assistant (KK), with a research background in nutrition and qualified as an overseas trained dental professional, was present during each of the focus groups. KK moderated the focus groups to ensure they were transparently conducted, and specifically reduce any bias with participants who were working with ZM and for the SND specialists, who knew ZM given the small number of SND specialists in Australia. Most interviewed participants were working at different sites to ZM within the public oral health service and were unaware of the research prior to participation. All interviewed SND specialist participants were working at different sites to ZM in New South Wales including interstate in Victoria, Queensland and South Australia. Participants not working in SND were unaware of the SND specialist role in managing people living with severe obesity prior to the research being conducted.

Focus groups and the two semi-structured interview recordings were transcribed verbatim by transcription software Trint™ 2022, Trint Limited, United Kingdom (UK). There were no requests for transcript review by participants, so transcripts were not returned to participants for comments and/or corrections. A thematic (inductive) analysis approach was undertaken to interpret the data and sort into themes and subthemes.[10] An initial coding framework was developed after two researchers (ZM, KK) independently coded two transcripts (10% of the data), with consensus checking then conducted with KM who did not conduct any of the interviews. Coding was performed using Quirkos 2.5.3 qualitative analysis software.

## Results

Thirty-four participants were recruited across clinician and support staff groups (Table 1). Five focus groups of between 2–3 participants each were conducted with support staff, including dental assistants (*n* = 9) and reception staff (*n* = 2). Seven focus groups of between 2–3 participants were carried out with clinicians, including general dentists (*n* = 8), oral health therapists (*n* = 5) and SND specialists (*n* = 8) in addition to the semi structured interviews with general dentists (*n* = 2).

**Table 1:** Demographics of participants by gender, role, employment type and lived experience with obesity

Participant characteristics	*N *= 34 (%)
Female	27 (79.4)
Male	7 (20.6)
*Role*	
General dentist	10 (29.4)
Oral Health Therapist	5 (14.7)
Specialist Special Needs Dentistry	8 (23.5)
Dental assistant	9 (26.5)
Dental receptionist	2 (5.9)
*Employment type*	
Public	23 (67.6)
Private	3 (8.8)
Mixed	8 (23.5)
*Self-reported lived experience with obesity*	
Yes	10 (29.4)

There were three key themes relating to observed experiences of weight stigma, weight stigma impacting on dental management, and stigma reduction strategies. Examples relating to each theme are included.

### Observed experiences of weight stigma in the dental setting

Numerous examples of verbal and non-verbal weight stigma were reported as being observed by some participants across dental settings, as well as more broadly. Specific to the dental setting, participants reported observing both verbal and non-verbal stigma, with the latter manifesting as looks of disgust.

*“You know, a lot of it is the body language that you can pick up. So it doesn't have to be verbal comments, but you know, just kind of people's perception. Whether it be in the dental setting or outside to see a patient who is obese then…as sad as it is, it's almost like look of disgust… and then just kind of, you know, turning away and just really not wanting to interact with that person” (SND specialist).*

Some participants felt that there may be more weight stigma in the broader community compared to dental settings. Very few participants reported having not experienced perceived weight stigma in the dental setting.

*“Look, when I think about the workplace, I don't feel there is (weight stigma) so…but I do feel it in life in general…that you know, people are judged differently. I'm not saying by me, but I mean in the community kind of thing…” (Dental assistant).*

When weight stigma did manifest in the dental setting, participants recognised weight stigma could be subtle, be individually dependent, or more widespread, although often concealed or influenced by social acceptance for example in scenarios behind the patient’s direct vision or before or after the patient attends the dental clinic.

*“It's really subtle, but it's ubiquitous, like the dental assistant will be coming into the room towards me. …and you can't see the patient yet and you know she rolls her eyes at me and puffs out her cheeks and then the patient comes in and obviously the patient is physically large and you think, what the hell were you saying? What was that body language supposed to communicate to me?…you know it's that sort of… immature behaviour it’s ubiquitous” (SND specialist).*

*“I guess…it just depends on the DA as well, so not just kind of saying everyone, I mean a lot of (people are) fantastic with it and they would be very empathetic” (Dental assistant).*

There were also reports of weight stigma from dental associated settings, such as hospital theatre environments, where dental treatment may be provided. One SND specialist participant related their experience of weight stigma in the dental setting to appraising this as a disappointing experience.

*“I think that they're always situations where it comes up where you just hear…there's just small comments…here and there and I guess, the most disappointing experience for me was treating a patient in theatre and you know, as soon as the patient is asleep…you're there focusing on the treatment that you need to be able to provide for this person. And you just hear these different sort of comments from different members of the nursing team, from the anaesthetic team about the patient being fat and all sorts of really terrible things. I mean, I think that it shows it.. really comes to fore in that type of situation” (SND specialist).*

### The impact of weight stigma on dental management

Whilst no participants reported explicit weight stigma to be a barrier to their dental management for people living with severe obesity, one participating clinician mentioned the possible psychosocial impacts of weight stigma on patients appraising dental disease preventive discussions and feeling judged.

*“I think from a more like emotional perspective, it's harder maybe sometimes when you're explaining what needs to occur and they're feeling a little bit, maybe more judgement when I'm discussing medical (history), when I'm discussing diet, when I'm discussing toothbrushing things like that, I think they sometimes have a concern that maybe they're being judged not only for their oral health but for their overall health…because people assume that they have a hole in their tooth. They assume that it's been because of sugary foods and that then it's linked with obesity” (Oral health therapist).*

### Stigma reduction strategies – an educational focus

Participants provided varied examples for stigma reduction strategies. For the SND specialists, these predominantly centred around advocacy for this patient group and education of their teams locally, the wider profession and medical colleagues regarding the category of bariatric patients that ideally should be referred for specialist management, with characteristics not based on weight assessment alone. Improving the appropriateness of referrals in this regard was perceived as being a measure to improve access and additionally reduce weight stigma associated with the current processes.

*“advocating within our dental team to reduce the barriers that patients might face …there's a need to advocate more broadly within our profession and outside our profession…for example, in the medical sphere, raising awareness … making them aware of the referral pathways …. and patients that we see” (SND specialist).*

*“with our training and with us being specialists in Special Needs Dentistry,…there's not that many of us and I feel like we need to be the ones that you know speak up and instil this change and hopefully has that flow on effect where we're reducing the stigma” (SND specialist).*

*“…the first point of interaction for a patient to experience, you know, to come and receive dental care is the auxiliary staff. They don't meet the dentist first. They meet the receptionist. They get a call from the receptionist. You know, asking the questions and getting them all the information and getting them to the dental surgery. And the dental assistant in my case, usually it's my dental assistant who brings them into the surgery for me. And so to me, I feel like that's part of our responsibility as clinicians is to make sure that every part of the patient's journey from the beginning to the end, …if we aren't speaking to the receptionist, who was the person who usually makes contact with them, we may not even get the opportunity to see that person turn up at our clinic” (SND specialist).*

Some general weight stigma reduction measures suggested included rapport building, taking the time to explore the patient’s story and creating a non-judgemental space.

*“Usually these patients share how they ended up having their obesity…there is always a story behind that we need to listen but seeing this patient in the general dentist community is quite difficult because we dedicate time to listen to those stories. If you have only 45 min to provide dental care then there is no time for that” (SND specialist).*

*“One of the ways to try to overcome this challenge or barrier is through trying to create that safe space and helping them realise that this is a non judgmental space” (SND specialist).*

There was a desire to have training for both clinicians and support staff and a team-based education approach recommended. Part of this suggested education for the dental team appeared to centre around weight discussions with people living with severe obesity and how these could be used to prevent further weight stigma. The majority of SND specialist participants conveyed an understanding of the need for tailored communication approaches for different members of the dental team.

*“I think there needs to be more education courses specifically to raise awareness and teach …making sure that they're tailored for an entire dental team and not just clinicians. Although having said that, there is very little exposure within undergrad and even within our postgraduate training course, I feel that it was a very underdeveloped topic.” (SND specialist).*

Support staff and oral health therapist participants felt their lived experiences of obesity could reduce weight stigma through an empathetic approach to patient care.

*“…Because of what I've been through myself. But I don't want that person to feel the way I did ever. I wanna treat her just like she's a human being, just like she is. And you know, every patient should be treated how we wanna be treated…or how we want our family to be treated not just …because they're on the pension or what have you, doesn't make them any different. Everyone’s got a story” (Dental assistant).
*

SND specialist participants reported the need for systemic change in addressing weight stigma in the dental setting.

*“Don't we need to be tackling it from a much wider point of view rather than, I guess thinking about from such a small group within our specialty? I'd like to see it to be something much more widespread” (SND specialist).*

## Discussion

The current study found several unique and previously unexplored perspectives of clinicians and support staff regarding weight stigma in the dental setting, in particular of SND specialists working in Australia. The inclusion of this range of participants with greater clinical experience in the dental management of people living with severe obesity as the differential factor, allowed for varied insights to be obtained from the focus groups conducted. A key finding was the wide-ranging manifestations of weight stigma in dental and related settings and its impact on dental management, particularly with regards to preventive dental management. Weight stigma in this study was broadly acknowledged to result in assumptions of patient’s oral health status with the potential to compromise dental care and negatively influence discussions with patients around their dental disease risk. Given weight stigma has been previously reported as a barrier to accessing dental services by people living with clinically severe obesity [5], this particular challenge requires further attention, particularly given the potential negative impacts of weight stigma on dental treatment provision as was reported in this study. These findings were similar to widespread consensus of the negative effects of weight stigma in general healthcare [11].

Numerous verbal and non-verbal examples were provided in both conventional and SND settings and differing views and perspectives surrounded participant awareness and experiences of weight stigma based on professional roles. Weight stigma was often experienced and observed by participants in settings where they were among professional colleagues within the dental team, without any reported action when it occurred or preventive measures to reduce stigma in the workplace. This was another unique finding of this study and reflects the complex drivers of weight stigma at both the individual and system level [12]. The inaction may be additionally reflective of the “otherness” concept, which describes where participants perceive no role of their own in contributing to weight stigma observed in others.

The key findings provide further impetus for the dental profession to participate in advocacy against the presence of weight stigma, both broadly and within local teams. This advocacy need was predominantly raised by SND specialist participants in this study which may be due to the considerable role they already play in disability discrimination advocacy within the dental and wider community. This suggests a need for further education regarding constructive responses and SND participants are best positioned to lead and guide educational interventions involving severe obesity and weight stigma in the dental setting.

Several weight stigma reduction strategies with an educational focus were proposed by dental team participants, contributing to previously unexplored data. Of significance, was the need for recognition of the underlying drivers of weight stigma to guide and develop interventions before they are implemented. Increased education has been identified as a weight stigma reduction strategy in healthcare settings and was further reinforced by participants in the current study [13, 14]. A recently proposed educational strategy involves the integration of an interactive storytelling environment against weight stigma which aims to break down multi-layered and self-perpetuating stigmatising attitudes and beliefs [15]. Educational interventions should be addressed early and continuously throughout healthcare or tertiary education and in clinical practice [13], a need emphasised by participants in this study and consistent with recommendations in the literature. Many participants expressed a clear desire for team based education approaches, given the important role that support staff play and potential for stigma experienced by people living with obesity across their entire patient journey, as was also a finding from previous qualitative data in the UK [6]. This team based education was suggested with a focus on communication and teamwork in managing the patient living with severe obesity, understanding their lived experience, and normalising weight discussions particularly when influencing patient safety, to reduce weight stigma. This would also enable the development of a team-based action plan to respond to observed experiences of weight stigma. The findings of this study suggest a need for revision of tertiary curricula and currently available continuing professional development on the topic of obesity and weight stigma within Australia, integrating the knowledge required by the dental team in the workplace.

### Strengths and limitations

There were some limitations to the current study given the predominantly female sample of non-specialist SND participants recruited from a single regional geographical distribution. The sample may therefore not have been representative of all dental clinicians and support staff. There may also have been sampling bias as participants with lived experience of obesity, or experience with managing patients living with obesity may have been more willing to participate in the study. Similarly, there may have been bias from the research team given the interest in the research topic by authors. The data is also limited by the inherently subjective nature of the qualitative interview data which may have contributed to an under-reporting of weight stigma experiences by participants.

However, this study also had some important strengths. The piloting of the interview schedule and semi-structuring of focus groups initially, ensured sensitivity in relation to this topic and that questions would be interpreted correctly. The focus group methodology employed was advantageous to elicit broad exploration of the topic of weight stigma [16]. The number of focus groups carried out was sufficiently high to have ensured key perspectives from both clinician and support staff groups. Another major strength of this qualitative study was the inclusion of perspectives of two groups, including general dental team members and SND specialists. In considering the perspectives of SND specialists nationally, given their prominent role in existing referral pathways across Australia, this study was able to elicit their unique insights from differing contexts and differing states. The paper also serves to bring awareness to the impact of weight stigma on dental management. To the authors’ knowledge, this is the first investigation of the perspectives of Australian dental clinicians and support staff on weight stigma. Given the differing context and limited access to SND services within Australia compared with for example, in the UK, the unique SND specialist considerations in this study provide previously unreported data.

## Conclusion

The current study explored observations of weight stigma in various dental settings which were described in depth with perspectives differing based on participant awareness and professional role. The negative impact of weight stigma on dental management was highlighted in assumptions of patient oral health status with the potential to compromise dental care and preventive dental discussions. Stigma reduction strategies need to target the identified barriers and address the complex drivers of weight stigma before implementation.

Findings of the current study emphasise the role for team-based education, led and guided by SND specialists through their professional advocacy roles and encourages the development of a dental team action plan to respond to observed experiences of weight stigma in the workplace.

## Supplementary Information

Supplementary Material 1.

## Data Availability

The datasets generated and/or analysed during the current study are not publicly available due to the sensitive nature of focus group discussions but are available from the corresponding author on reasonable request.

## Abbreviation

SND: Special Needs Dentistry
