# Supplementary material for: “Dental team-based education” Dental team perspectives and experiences about weight stigma: a qualitative analysis
Source: BMC Oral Health. 2025 Apr 6;25:493. doi: 10.1186/s12903-025-05854-1 (PMC11972485; doi:10.1186/s12903-025-05854-1)
Supplement: Supplementary file 1 — Supplementary Material 1. [file 12903_2025_5854_MOESM1_ESM.docx]

**Obesity STIGMA-DENT STUDY Focus group questions – support staff**

-Tell me about your experiences in interacting with adult patients with obesity in the workplace?

-How have you experienced asking adult patients questions around weight?

-How has the response been from patients in your opinion and does this influence your approach??

-What is your understanding of weight stigma? If weight stigma is described as the negative attitudes, beliefs or discrimination based on a person’s weight and/or body size, tell us about any experiences you have with this – personal or patient related? How might your workplace increase or decrease weight stigma?

-What if any areas would you wish to have further training with regards to obesity?

Closing question: Is there is anything else you would like to tell me about your thoughts on weight stigma experienced by adults living with obesity in the dental setting or by dental professionals?

Thanks for your time and participation.

**Obesity STIGMA-DENT STUDY Focus group questions – clinicians**

1.What is your understanding of adult obesity and what can be done about it? What do you feel is your role (if any) in the prevention or management of obesity?

SubQ1: How have you experienced asking adult patients questions around weight? How has the response been from patients in your opinion and does this influence your approach? Describe any differing experiences (if any) when managing adult compared with paediatric patients with obesity? Why do you believe this might be different?

SubQ2: Compared with your usual preventive oral healthcare advice (including diet and oral hygiene) are there any changes you would make to the preventive advice you would give to a patient with obesity? Do links between obesity and periodontal disease or caries form part of your preventive discussions with patients with obesity?

2.What kind, if any, barriers might adults with obesity be facing in your workplace?

SubQ 1: What is your understanding of weight bias or stigma? If weight stigma is described as the negative attitudes, beliefs or discrimination based on a person’s weight and/or body size, tell us about any experiences you have with this – personal or patient related? How might your workplace increase or decrease weight stigma? How do you think it is best to raise the issue of weight amongst people with obesity without being stigmatising?

SubQ 2: What kind of effects might this weight stigma have for people with obesity?

Sub Q3: What differences with regards to weight stigma might adults compared with children face in your workplace?

3. Tell me about any education and training relating to the dental management of adults with obesity you have been exposed to eg university training at undergraduate or post graduate level, CPD courses

Sub Q1: - What if any areas would you wish to have further training with regards to obesity?

Sub Q2: What areas would be beneficial for you to be included in guidelines on adult obesity management for dental professionals?

Closing question: Is there is anything else you would like to tell me about your thoughts on weight stigma experienced by adults living with obesity in the dental setting or by dental professionals?

Thanks for your time and participation.
